# Knowledge, attitude and practice of cervical cancer screening and associated factors amongst female students at Wollega University, western Ethiopia

**DOI:** 10.1186/s13104-019-4564-x

**Published:** 2019-08-19

**Authors:** Temesgen Tilahun, Tamirat Tulu, Worku Dechasa

**Affiliations:** 1 Institute of Health Sciences, Wollega University, Nekemte, Ethiopia; 2 East Wollega Zone Health Office, Nekemte, Ethiopia

**Keywords:** Cervical cancer, Screening, Associated factors

## Abstract

**Objective:**

This study aimed to assess the knowledge, attitude and practice of cervical cancer screening and associated factors among female students of Wollega University, western Ethiopia in 2017.

**Results:**

More than half, 54.4%, of participants had heard about cervical cancer and its risk factors. Only 35.8% knew about screening procedures such as pap smear (61.1%) and visual inspection with application of acetic acid (38.8%). Study participants from age of 21 to 25 years were about six times more likely to have satisfactory knowledge on cervical cancer screening compared to other age groups. Less than half of the study participants (44.1%) had a positive attitude towards cervical cancer screening. None of the study participants had been screened for cervical cancer in the past 3 years. Thus, different governmental and nongovernmental stakeholders need to focus on awareness about cervical cancer, its screening and preventive strategies.

## Introduction

Cervical cancer is a cancer of uterine cervix. It is the second most common cancer in women worldwide and the leading cause of cancer deaths in developing countries. It is almost always associated with human papilloma virus (HPV) infection. In addition to this infection, factors including multiparty, smoking, prolonged use of oral contraceptives, low socioeconomic status, sexual transmitted infections, 1st sexual intercourse at younger age, low immune status and factors related to poverty are associated with cervical cancer [1, 2].

Cervical cancer is said to be preventable disease. One reasons for this is due to the availability of effective screening options such as Pap smear, HPV-DNA testing and visual inspection with application of acetic acid and iodine [3]. However, knowledge, attitude and practice of cervical cancer screening is low because of numerous factors [4, 5].

Because of its high burden and high mortality, cervical cancer is considered a priority cancer for intervention. Ethiopia has put in place a strategic goal to reduce cancer incidence and mortality by 15% by 2020 [3]. Female students in particular can play an important role beyond recommending the uptake of HPV vaccines; they can be a source of information for female relatives, parents, teachers, health care professionals, and the wider community [4].

Despite the growing number of cervical cancer cases in Ethiopia, there is still a gap in knowledge, attitude and practice of cervical cancer screening [6, 7].

It is clear that cervical cancer incidence peaks in older women. This study focused on University students because they are the future leaders of tomorrow’s generations and are responsible for increasing awareness about cervical cancer and screening. It is therefore crucial to assess their base line knowledge, attitude and practice of cervical cancer screening.

## Main text

### Methods and materials

The study was conducted in Wollega University from September 28, 2017 to October 30, 2017. The University is a public higher educational institution established in February 2007. Currently, the university teaches undergraduate and postgraduate students with campuses in Nekemte, Gimbi and Shambu. The study design was an institution based cross-sectional study.

From all Wollega university undergraduate female students, aged more than 17 years, who were registered for the academic year 2016/17, sample size was calculated by using single-population proportion formula based on the following assumption. Proportion of female student having knowledge about cervical cancer Screening 56.8% (P = 0.568) (9), design effect 2, and considering 95% confidence interval, and margin of error to be 5% (d = 0.05) and 10% non-response rate. The sample size was therefore calculated to be 830. Selection of study participants was done using simple random sampling methods.

A pre-tested structured questionnaire was developed for data collection, after reviewing similar literatures. Six health professionals were recruited and trained to collect and fill data, and on issues related to the confidentiality of the response and rights of the participants. Data on sociodemographic characteristics and different variables related to cervical cancer screening were collected. All completed questionnaires were checked for completeness by the principal investigator.

The data were entered, cleaned and analyzed by using SPSS for windows version 20.0. Descriptive analysis using frequency, mean, median, standard deviation and percentages was performed. Chi-square test was used to assess the association between knowledge and attitude, and knowledge and practice. Bivariate analysis was done by cross tabulation of each independent variable with the outcome to look at the existence of significant associations and to determine adjusted odds ratio (AOR). Multivariate logistic regression model was used to control the effect of confounding variables. All variables having P < 0.25 in the bivariate analysis were included in multivariable logistic regression analysis. The results of the final model were expressed in terms of odd ratio (OR) and 95% confidence intervals (CI) statistical significance was defined by a P-value of less than 0.05.

Regarding knowledge, attitude and practice of cervical cancer screening, we made the following definitions. For questions assessing knowledge, a score of one and zero were given for correct and incorrect responses respectively. For each area of knowledge, the scores of the responses were summed-up and the total divided by the number of questions, giving a mean score. These scores were converted into a percentage. Knowledge was considered ‘satisfactory’ if the percent score was 60% or more and ‘unsatisfactory’ if less than 60%.

Attitude towards cervical cancer screening was measured using a 5-point Likert scale as individuals responding strongly with a favorable attitude were given a score of 5. 1 was allocated to those who responded strongly in disagreement. The scoring was reversed when asking questions around unfavorable attitudes. The scores of the items was summed-up and the total divided by the number of the items, giving a mean score. These scores were converted into a percentage, and the means and standard deviations were computed. The attitude was considered ‘favorable’ if the percent score was 60% or more and ‘unfavorable’ if less than 60%.

The practice was assessed by asking respondents if they had attended a screening test for precancerous lesions at least once or if they had not attended in the past 3 years. Those who had attended screening were considered to be following practice. Those who had not attending screening were considered not to be following practice.

### Results

#### Socio-demographic characteristics of study participants at Wollega University

Of 830 female students at Wollega University who were invited to complete the questionnaires, a total of 805 students completed the questionnaire, a response rate of 96.9%. The age range of participants were 17 to 26 years with a mean age of 22.8 years. The majority of participants were from the school of engineering (42.7%) and were 2nd year students (61.4%) (Table 1).

**Table 1:** Socio-demographic characteristic of respondents at Wollega University, 2017

Variables	Frequency	Percent
Age in years		
15–20	25	3.1
21–25	675	83.9
26–30	105	13.0
15–20	25	3.1
Marital status		
Married	36	4.5
Single	769	95.5
Religion		
Orthodox	167	20.7
Protestant	480	59.6
Muslim	107	13.3
Catholic	51	6.3
Ethnicity		
Oromo	608	75.5
Amhara	143	17.8
Tigre	12	1.5
Others	42	5.2
School/college		
Engineering and technology	344	42.7
Natural and computational sciences	159	19.8
Medicine and health sciences	91	11.3
Business and economics	145	18.0
Social sciences and humanities	66	8.2
Year of study		
Second year	494	61.4
Third year	159	19.8
Forth year	127	15.8
Fifth year	25	3.1

#### Knowledge of participants on cervical cancer and its screening

More than half, 54.4%, of participants had heard about cervical cancer and its risk factors. 37.9%, 49.5% and 12.5% of study participants believed that having many sexual partners, early initiation of sexual intercourse and human papilloma virus respectively were the major risk factors for cervical cancer. When asked about methods to prevent cervical cancer, they reported avoidance of multiple sexual partner (54.8%), prevention of infection with HPV (24.5%) and use of condom during sexual intercourse (20.7%). Only 6.3% of the participants reported that cervical cancer can cause death. The sources of information about cervical cancer were mass-medias such as television and radio (36%), brochures and posters (12.4%), health workers (7.3%) and teachers (0.7%).

Only 35.8% of participants knew about screening procedures like Pap smear (61.1%) and visual inspection with application of acetic acid (38.8%). Sources of information on screening procedures were mass-medias like TV and radio (96.1%), reading different books (3.5%) and teachers (0.34%). The above findings indicate that knowledge level of study participants on cervical cancer and its screening were unsatisfactory. For instance, 60.49% of study participants had unsatisfactory knowledge on cervical cancer screening.

#### Association between socio-demographic characteristics and knowledge about cervical cancer screening

Year of study and college/school were not associated with satisfactory knowledge on cervical cancer screening in multivariate analysis. Students/participants aged 21 to 25 years were six times more likely to have satisfactory knowledge compared to other age groups (AOR = 5.8, 95% CI 1.9–18) (Table 2).

**Table 2:** Association between socio-demographic characteristics and knowledge score of cervical cancer screening among study participants at Wollega University, 2017

Characteristic	Category	Knowledge status		COR 95% CI	AOR 95% CI
		Unsatisfactory knowledge	Satisfactory knowledgeable		
Age in years	15–20	21 (4.3%)	4 (1.3%)	1	
	21–25	361 (74.1%)	314 (98.7%)	4.56 (1.55,13.44)	5.8 (1.9,18)
	26–30	105 (21.6%)	0 (0%)		
Year of study	Second year	265 (54.4%)	229 (72%)	1	
	Third year	123 (25.3%)	36 (11.3%)	0.33 (0.22, 0.51)	0.84 (0.5, 1.4)
	Forth year	80 (16.4%)	47 (14.8%)	0.68 (0.45, 1.01)	0.89 (0.56, 1.41)
	Fifth year	19 (3.9%)	6 (1.9%)	0.36 (0.14, 0.93)	0.9 (0.63, 1.8)
College/school	Engineering and technology	197 (22%)	147 (46.2%)	1	1
	Natural and computational sciences	107 (22%)	52 (16.4%)	0.65 (0.44, 0.96)	1.1 (0.69, 1.78)
	Medicine and health Sciences	55 (11.3%)	36 (11.3%)	0.87 (0.54, 1.4)	1.3 (0.74, 2.2)
	Business and economics	80 (16.4%)	65 (20.4%)	1.09 (0.73, 1.61)	0.99 (0.64, 1.5)
	Social Science and humanities	48 (9.9%)	18 (5.7%)	0.5 (0.28, 0.9)	0.73 (0.37, 1.4)

#### Attitude of study participants about screening of cervical cancer

Less than half (44.1%) of study participants had a positive attitude towards cervical cancer screening. Only 10.6% and 16% of the participants agreed on the importance of a national screening program for precancerous lesions and HPV vaccination to prevent cervical cancer respectively. Year of study and age of participants were significantly associated with positive attitude. Third year students (AOR = 1.7, 95% CI 1, 2.8) and participants in the age group 21 to 25 years (AOR = 12.7, 95% CI 3.9, 4.9) were approximately 0.7 times and 11.7 times more likely to have a positive attitude towards cervical cancer screening when compared to their counterparts (Table 3).

**Table 3:** Socio-demographic characteristics and attitude towards cervical cancer screening among study participants at Wollega University, 2017

Variables	Category	Attitude status		COR 95% CI	AOR 95% CI
		Positive attitude	Negative attitude		
Age in years	15–20	13 (3.7%)	12 (2.7%)	1	
	21–25	243 (68.5%)	432 (96%)	0.51 (0.23, 1.15)	0.47 (1.9, 1.1)
	26–30	99 (27.9%)	6 (1.3%)	15.2 (4.8, 47.5)	12.7 (3.9, 41.9)
Year of study	Second year	184 (51.8%)	310 (68.9%)	1	
	Third year	108 (30.4%)	51 (11.3%)	3.5 (2.4, 5.2)	1.7 (1.0, 2.8)
	Forth year	51 (14.4%)	76 (16.9%)	1.1 (0.75, 1.6)	0.8 (0.5, 1.3)
	Fifth year	12 (3.4%)	13 (2.9%)	1.5 (0.69, 3.4)	0.7 (0.2, 1.9)
College/school	Engineering and technology	138 (39.9%)	206 (45.8%)	1	
	Natural and computational sciences	76 (21.4%)	83 (18.4%)	1.36 (0.93, 1.99)	0.8 (0.49, 1.3)
	Medicine and health sciences	46 (13%)	45 (10%)	1.5 (0.95, 2.4)	1.0 (0.59, 1.8)
	Business and economics	61 (17.2%)	84 (18.7%)	1.0 (0.73, 1.6)	1.2 (0.77, 1.9)
	Social science and humanities	34 (9.6%)	32 (7.1%)	1.5 (0.93, 2.7)	0.69 (0.66, 2.5)

#### Practice towards screening for cervical cancer

None of the study participants had been screened for cervical cancer in the past 3 years.

### Discussion

This study was conducted to assess the knowledge, attitude and practice of cervical cancer screening and its associated factors among female students at Wollega University. This research showed that 54.4% of female students at Wollega University had heard about cervical cancer. This is higher than a finding in Nigeria (15%) and Cameroon (28%) of respondents had heard about cervical cancer [9, 10]. However, our finding was lower when compared to a similar study in Ukraine among female medical students of Crimea State Medical University where 80% of participants had heard about cervical cancer [11]. This gap might be due to differences in awareness amongst study participants in these studies. Of concern, only 38.3% of the study participants in this study knew about cervical cancer screening methods. This needs special attention as identification of precancerous lesions is crucial for early treatment and prevention progression to cervical cancer.

In this study, 54.4% of study participants had heard about risk factors of cervical cancer. This is lower than similar studies amongst female medical students of Crimea State Medical University in Ukraine [11], female university graduates in Bhutan [12] and female students of Hawassa University in Ethiopia [8]. Increasing knowledge, by different means, on risk factors could have direct effect on the prevention of cervical cancer.

According to this study, there was a significant association between the age of participants and their knowledge level regarding cervical cancer screening. Participants aged 21 to 25 years were approximately six times more likely to have satisfactory knowledge compared to other age groups. Thus, attention should be given to the other age groups to raise their knowledge level regarding cervical cancer screening.

In this study, only 44.1% of the study participants had a positive attitude towards cervical cancer screening. This is much lower than a similar study conducted in Hawassa University among female students [8]. Surprisingly, only 10.6% of the participants agreed on importance of a national screening program for precancerous lesions. Similarly, only 16% of participants agreed on the importance of HPV vaccination to prevent cervical cancer. This might be due to a lack of adequate information about the severity of cervical cancer national policy in Ethiopia. Age and year of study of participants were identified as factors affecting a positive attitude. For instance, 3rd year students/participants had a positive attitude towards cervical cancer screening twice that of other batches. In addition, participants aged 21 to 25 years were 13 times more likely to have a positive attitude towards cervical cancer screening when compared to other age groups.

Surprisingly, none of the study participants had undergone cervical cancer screening. This is explained by lack of or limited access to screening services, and low level of knowledge and attitude among the study participants. Thus, different sectors should give priority attention for younger age group so as to prevent them from cervical cancer.

### Conclusion

In conclusion, knowledge, attitude and practice regarding cervical cancer screening among female students at Wollega University were low and thus the knowledge and attitude amongst the general population is likely even to be lower. Thus, different governmental and nongovernmental stakeholders need to give special attention on raising awareness about cervical cancer, its screening and preventive strategies.

## Limitations

This study was a cross-sectional study and may not show the cause and effect relationship. The other limitation could be small sample that might lead to statistical imprecision.

## Data Availability

The data sets used and analyzed during the current study are available from the corresponding author on reasonable request.

## Abbreviations

CI: confidence interval
AOR: adjusted odd ratio
SPSS: Statistical Package for Social Sciences
HPV: human papilloma virus

## References

[1] Cancer incidence, mortality and prevalence worldwide. International Agency for Research on Cancer, 2012. Available at http://globocan.iarc.fr. Accessed 3 Mar 2017.

[2] UNFPA. Comprehensive cervical cancer prevention and control program guidance for countries; 2011.

[3] Loutfi A, Pickering JL (1992). The distribution of cancer specimens from two pathology centers in Ethiopia. Ethiop Med J.

[4] WHO, United Nations, The World Bank, IARC Globocan. Cervical cancer crisis card, 2013. Available at http://www.who.int/hpvcentre/statistics/en/. Accessed 3 Mar 2017.

[5] Waktola EA, Mihret W, Bekele L (2005). HPV and burden of cervical cancer in East Africa. Gynecol Oncol.

[6] Akinwuntan AL (2008). Correlation of cervical cytology and visual inspection with acetic acid in HIV-positive women. J Obstet Gynaecol.

[7] Terefe Y, Gaym A (2008). Knowledge, attitude and practice of screening for carcinoma of the cervix among reproductive health clients at three teaching hospitals, Addis Ababa, Ethiopia. Ethiop J Reprod Health.

[8] Tsegaye S. Knowledge, attitude, practice of cervical cancer screening and its associated factors among female students in Hawassa University College of medicine and health science Hawassa Ethiopia. Available at http://www.hu.edu.et/hu/images/pdf/proceedings/. Accessed 3 Mar 2017.

[9] Ahmedin J (2011). Global cancer statistics. CA Cancer J Clin.

[10] Tebeu PM (2008). The attitude and knowledge of cervical cancer by Cameroonian women; a clinical survey conducted in Maroua, the capital of Far North Province of Cameroon. Int J Gynecol Cancer.

[11] Global cancer facts & figures 3rd edition. Available at https://www.cancer.org/.../cancer.../cancer-facts.../global-cancer-facts...figures/global. Accessed 3 Mar 2017.

[12] Dhendup T, Tshering P (2014). Cervical cancer and screening behaviours among female university graduates of year 2012 attending national graduate orientation program, Bhutan. BMC Women’s Health.

